# Assurance of Timely Access to Breast Cancer Diagnosis and Treatment by a Regional Breast Health Clinic Serving Both Urban and Rural-Remote Communities

**DOI:** 10.3390/curroncol30010095

**Published:** 2023-01-16

**Authors:** Elizabeth Ewart, Anise Barton, Leo Chen, Ross Cuthbert, Kaitlin Toplak, Andrea Burrows

**Affiliations:** Department of Surgery, The University of British Columbia, 2775 Laurel Street, 11th Floor, Vancouver, BC V5Z 1M9, Canada

**Keywords:** breast cancer, wait times, diagnosis, treatment, surgery, delivery of care, rural, remote

## Abstract

In response to breast cancer diagnostic regional wait times exceeding both national and provincial standards and to symptomatic patient referrals for diagnostic mammography taking longer than abnormal screening mammography referrals, the Rae Fawcett Breast Health Clinic (RFBHC) was opened in 2017 in a mid-sized Canadian hospital serving both urban and rural-remote communities. We investigated whether the RFBHC improved wait times to breast cancer diagnosis, improved compliance with national and provincial breast cancer standards, and decreased the wait time disparity associated with referral source. Statistical analyses of wait time differences were conducted between patients who were diagnosed with breast cancer prior to and after the RFBHC establishment. Study group compliance with national and provincial standards and wait time differences by referral source were also analysed. A survey was administered to assess overall patient experience with the RFBHC and clinic wait times. RFBHC patients had a shorter mean wait to breast cancer diagnosis (24.4 vs. 45.7 days, *p* ≤ 0.001) and a shorter mean wait to initial breast cancer treatment (49.1 vs. 78.9 days, *p* ≤ 0.001) than pre-RFBHC patients. After the RFBHC establishment, patients who attended the RFBHC had a shorter mean wait time to breast cancer diagnosis (24.4 vs. 36.9 days, *p* = 0.005) and to initial treatment (49.1 vs. 73.1 days, *p* ≤ 0.001) than patients who did not attend the clinic. Compliance with national and provincial breast cancer standards improved after the RFBHC establishment and the wait time disparity between screening mammography referrals and symptomatic patient referrals decreased. Survey results indicate that the RFBHC is meeting patient expectations. We concluded that the establishment of a breast health clinic in a Canadian center serving urban and rural-remote communities improved breast diagnostic services.

## 1. Introduction

The diagnosis and treatment of breast cancer is highly interdisciplinary and involves multiple processes including screening, diagnostics, treatment, and follow-up. Decentralized management of these processes contributes to unnecessary delays in breast cancer diagnosis and treatment, resulting in worsened clinical outcomes, increased downstream treatment cost, and heightened stress and anxiety for patients [1,2,3,4,5,6,7,8,9,10,11,12,13,14,15,16].

To address these challenges, breast health clinics (BHCs) dedicated to the centralized delivery of comprehensive breast cancer diagnostic and treatment services have been established in Europe [17,18,19,20,21], the US [22,23], and Canada [1,24,25,26,27,28,29]. Models for coordinated breast cancer care vary considerably between BHCs [29,30,31,32], but in Canada and abroad they have achieved reduced wait times when compared to usual care [24,25,26,27,28,29,30,31,32,33,34]. While limited evidence is available, studies demonstrate that BHCs may be especially impactful for rural-remote communities due to the financial and emotional burdens associated with travel to access care [35,36,37,38,39,40,41,42,43,44,45,46,47,48,49,50].

In May 2017, the Rae Fawcett Breast Health Clinic (RFBHC) was opened to address a growing need for coordinated breast cancer diagnostic services in Kamloops, British Columbia and its surrounding region. In collaboration with the Medical Imaging Department at the Royal Inland Hospital (RIH), the RFBHC serves the Thompson-Cariboo-Shuswap region, with a total population of approximately 251,000 distributed across 120,157 square kilometers (two people per square kilometer). This region includes the Kamloops urban community (population 129,286), as well as many rural-remote communities [51]. Prior to the establishment of the RFBHC, this region was not meeting national or provincial wait-time standards for breast cancer diagnoses and treatment. Additionally, patients referred for breast diagnostic imaging via the Screening Mammogram Program (SMP) were inadvertently prioritized over patients referred by their primary care provider (PCP) for a breast cancer symptom.

An objective of the RFBHC was to improve breast cancer wait times in alignment with national and provincial standards by implementing the Triple Assessment in one day Model (TAM) [52]. This model schedules, in a single appointment, (1) nurse and physician assessment, (2) breast diagnostic imaging with digital mammography and/or targeted breast ultrasound, and (3) (when indicated) ultra-sound guided core needle breast biopsy. The TAM was adapted from European centers and has been validated by breast clinics in Toronto and Vancouver, both reporting a significant reduction in wait time from presentation to breast cancer diagnosis after implementation [29,30].

The RFBHC is staffed by family physicians who have completed extra training in benign and malignant breast disease. Additionally, the clinic has dedicated clerical staff and a nurse navigator to coordinate patient care with diagnostic imaging, follow-up, breast cancer counselling, and referrals for treatment. The RFBHC submits surgical referrals for breast cancer treatment through a central surgical referral service which was independently established at the same time as the clinic and ensures patients receive surgical consultation in a timely manner. Oncology referrals are submitted to our local medical oncology team comprising three general medical oncologists supported by general practitioners in oncology. Generous public donations in support of the RFBHC enabled the expansion of the RIH Medical Imaging Department with an additional 0.5 full-time equivalent US technician and 0.5 full-time-equivalent mammography technician. This allowed the implementation of new triaging guidelines aimed at improving overall access to diagnostic breast imaging and decreasing the disparity between SMP and PCP diagnostic imaging referral times.

The purpose of this study was to evaluate the effect of the RFBHC on the following measures: (1) wait times within the breast cancer diagnosis and treatment pathway; (2) compliance with national and provincial breast cancer diagnosis and treatment standards; and (3) disparity between breast cancer diagnostic wait times for women presenting with an abnormal screening mammogram compared to women presenting with a symptom of breast cancer. A patient experience survey was administered to assess whether patient expectations were being met. Results of the study have been used for quality improvement within the RFBHC and may inform system change to breast diagnostic services in other Canadian centers serving rural-remote communities.

## 2. Methods

This was a retrospective cohort study approved by the UBC Research Ethics Board and the Interior Health Research Ethics Board. The study was completed at the Rae Fawcett Breast Health Clinic in Kamloops, British Columbia. A chart analysis of all women who had breast cancer surgery at Royal Inland Hospital (*n* = 539) between 1 January 2015 and 30 April 2019 was performed. Supplementary data were accessed from surgical offices in Kamloops. All women diagnosed with Ductal Carcinoma in Situ (DCIS), or invasive ductal or lobular carcinoma at Royal Inland Hospital during the study period, and who were over 18 years of age and received curative intent treatment, were included in the study. Patients with incomplete data, non-malignant breast lesions, or who were treated with palliative intent were excluded from this study. Data collection included wait times across key intervals of the breast cancer diagnosis and treatment pathway, age, home address (inside or outside City of Kamloops), referral source (SMP or PCP), and diagnosis.

**Figure 1:**
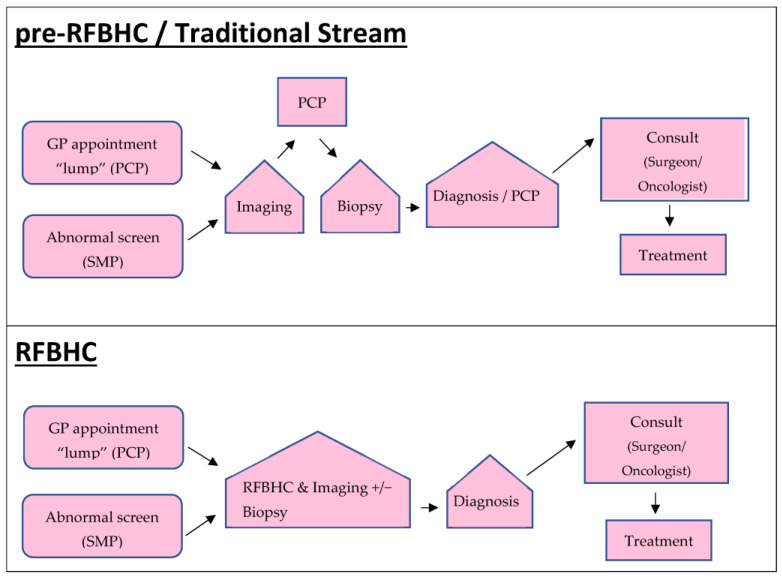
Diagnostic pathways. Pre-RFBHC patients were diagnosed with breast cancer prior to the establishment of the RFBHC (i.e., 1 January 2015 to 30 May 2017). RFBHC and Traditional Stream patients were diagnosed 30 May 2017 to 30 April 2019.

Patients were divided into three study groups: (1) those who received their breast cancer diagnosis before the RFBHC establishment (pre-RFBHC); (2) those who received their breast cancer diagnosis through the RFBHC; and (3) those who received their breast cancer diagnosis after the RFBHC establishment but remained in the Traditional Stream of PCP-coordinated care/did not attend the RFBHC. The RFBHC group was compared to the pre-RFBHC group and to the Traditional Stream group to assess how the RFBHC impacted breast cancer wait times for patients who attended it. The Traditional Stream group was compared to the pre-RFBHC group to determine if the RFBHC had a broader systemic impact on breast cancer wait times. Figure 1 outlines the pathway for each study group. Patients in the RFBHC group were offered a “triple assessment in one day” (TAM), while patients in the pre-RFBHC and the Traditional Stream groups had investigations and ongoing care coordinated by a PCP.

**Figure 2:**
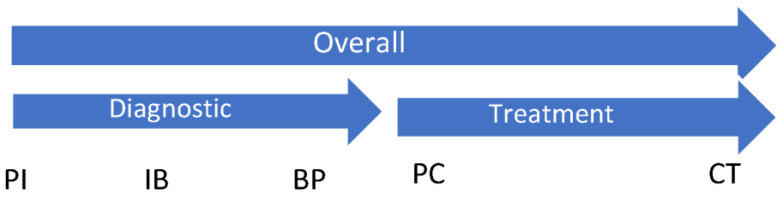
Study primary endpoints: Overall (presentation to initial treatment), Diagnostic (presentation to pathology), Treatment (pathology to initial treatment). Study secondary endpoints: PI (presentation to imaging); IB (imaging to biopsy); BP (biopsy to pathology); PC (pathology to specialist consult); and CT (consult to initial treatment).

The primary study endpoint was time from presentation to initial breast cancer treatment—surgical or neoadjuvant chemotherapy, whichever occurred first (Overall). This overall endpoint was further divided into time from presentation to pathology (Diagnostic) and time from pathology to initial treatment (Treatment). Secondary endpoints included times from presentation to diagnostic imaging (PI), imaging to biopsy (IB), biopsy to pathology (BP), pathology to specialist consult (PC), and consult to initial treatment (CT). Wait times across key intervals were compared between study groups and evaluated for accordance with published national and provincial standards. Figure 2 illustrates the study endpoints.

Wait time differences between groups were evaluated using linear regression analysis. *p*-values for compliance with national and provincial standards were evaluated using logistic regression. Mean overall wait times for each study group based on referral source (SMP vs. PCP) were evaluated using two sample *t*-tests. The level of significance for all statistical tests was established as *p* < 0.05. All analyses were conducted in R (version 4.0.2). Wait time standards were taken from the Pan-Canadian Standards for Breast Cancer Surgery [53] (national), the Canadian National Breast Cancer screening strategy [54] (provincial current), and the 2012 BC Provincial Breast Health Strategy Summary Report [55] (provincial recommended). Table 1 summarizes the wait time standards.

Patient experience with the RFBHC was evaluated with an anonymous questionnaire, modified from the Health Quality Ontario Primary Care experience survey [56,57], given to patients attending the clinic between September and December 2020. Two questions were asked: (1) “Thinking of your most recent visit, how would you rate the length of time it took between your referral/screening mammogram to the time you were first seen at the clinic?”; and (2) “Thinking of your most recent visit, how would you rate your overall experience?” Respondents chose from 5 responses: (1) Poor; (2) Fair; (3) Good; (4) Very good; and (5) Excellent.

## 3. Results

Of 539 patients who had breast cancer surgery between 1 January 2015 and 30 April 2019, 327 (61%) were diagnosed prior to RFBHC establishment (pre-RFBHC) and 212 (39%) were diagnosed after RFBHC establishment. Of those diagnosed after RFBHC establishment, 108 (51%) attended the RFBHC and 104 (49%) received care through the Traditional Stream. Table 2 summarizes patient and referral characteristics for each cohort.

**Figure 3:**
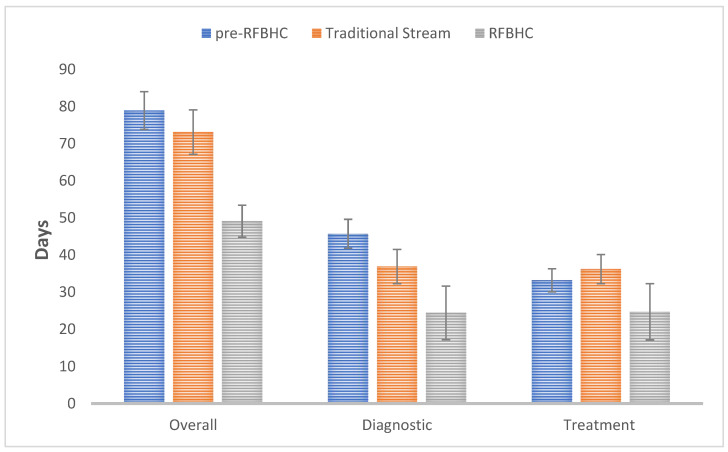
Primary mean wait time endpoints: Overall (presentation—initial treatment), Diagnostic (presentation-pathology), and Treatment (pathology—initial treatment). Whiskers represent 95% Confidence Intervals.

Figure 3 illustrates the primary mean wait times experienced within each study group:

- Compared to the pre-RFBHC group, the RFBHC group had a 29.8 days shorter mean overall wait time (49.1 vs. 78.9 days, *p* ≤ 0.001), 21.3 days shorter mean diagnostic wait time (24.4 vs. 45.7 days, *p* ≤ 0.001), and 8.5 days shorter mean treatment wait time (24.7 vs. 33.2 days, *p* = 0.037).
- After the establishment of the RFBHC and compared to the Traditional Stream group, the RFBHC group had a 24.0 days shorter mean overall wait time (49.1 vs. 73.1 days, *p* ≤ 0.001), 12.5 days shorter mean diagnostic wait time (24.4 vs. 36.9 days, *p* = 0.004), and 11.5 days shorter mean treatment wait time (24.7 vs. 36.2 days, *p* = 0.007).
- Compared to the pre-RFBHC group, the Traditional stream group had an 8.8 days shorter mean diagnostic wait time (36.9 vs. 45.7 days, *p* = 0.004), but no significant differences were found in overall wait time (73.1 vs. 78.9 days, *p* = 0.146) or treatment wait time (36.2 vs. 33.2 days, *p* = 0.220).

**Figure 4:**
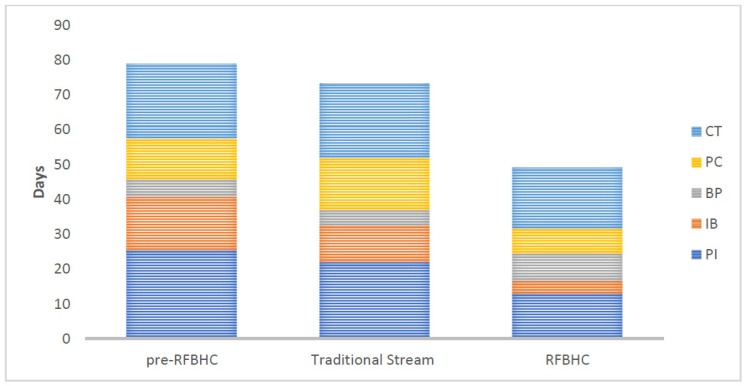
Secondary mean wait-time endpoints: presentation to diagnostic imaging (PI), diagnostic imaging to biopsy (IB), biopsy to pathology (BP), pathology to consult (PC), and consult to initial treatment (CT) for each study group.

Figure 4 illustrates secondary endpoints within each group:

- Compared to the pre-RFBHC group, the RFBHC group had on average: 12.4 days shorter wait time from presentation to imaging (13.0 vs. 25.4 days, *p* ≤ 0.001), 11.7 days shorter wait time from imaging to biopsy (3.6 vs. 15.3 days, *p* ≤ 0.001), and 4.0 days shorter wait time from consult to treatment (17.5 vs. 21.5 days, *p* = 0.019). No significant differences were found in wait times from biopsy to pathology (7.8 vs. 5.0 days, *p* = 0.415) or from pathology to consult (7.2 vs. 11.7 days, *p* = 0.228).
- After the RFBHC establishment and compared to the Traditional Stream group, the RFBHC group had on average: 9.0 days shorter wait time from presentation to imaging (13.0 vs. 22.0 days, *p* ≤ 0.001), 6.9 days shorter wait time from imaging to biopsy (3.6 vs. 10.5 days, *p* ≤ 0.001), 7.7 days shorter wait time from pathology to consult (7.2 vs. 14.9 days, *p* = 0.045), and 3.8 days shorter wait time from consult to treatment (17.5 vs. 21.3 days, *p* = 0.042). Wait time differences from biopsy to pathology were not statistically significant (7.8 vs. 4.4 days, *p* = 0.320).
- Compared to the pre-RFBHC group, the Traditional stream group had on average: 4.8 days shorter wait time from imaging to biopsy (10.5 vs. 15.3, *p* ≤ 0.001) and 3.2 days longer wait time from pathology to consult (14.9 vs. 11.7 days, *p* = 0.040). No statistically significant differences were found from presentation to imaging (22.0 vs. 25.4, *p* = 0.190), biopsy to pathology (4.4 vs. 5.0, *p* = 0.094), or consult to treatment (21.3 vs. 21.5, *p* = 0.901).

Compared to the pre-RFBHC group, the RFBHC group had improved statistical compliance with 4 of the 5 provincial and national breast cancer wait time standards included in this study (Table 3). Conversely, compared to the pre-RFBHC group, the Traditional Stream group had poorer compliance with the Pan-Canadian Standard for wait time from pathology to consult. No other statistically significant differences in wait time standards were identified between the pre-RFBHC and Traditional stream groups.

**Figure 5:**
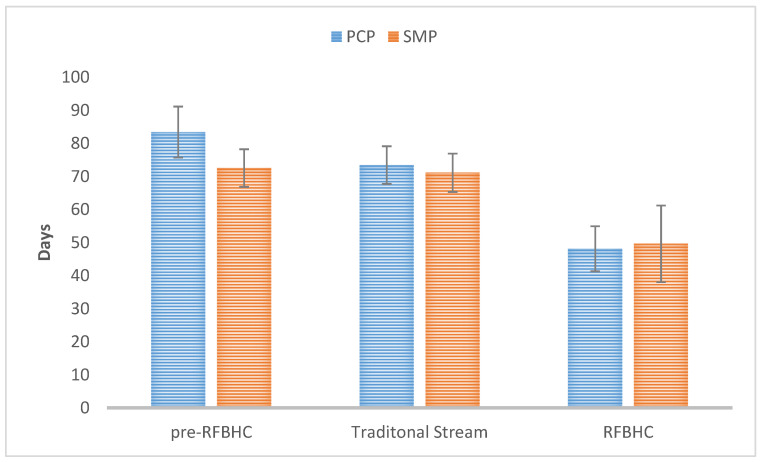
Overall wait time by referral source for each group. Whiskers represent 95% Confidence Intervals.

In the pre-RFBHC group, patients referred through the Screening Mammography Program (SMP) had on average 10.9 days shorter overall wait time (72.5 vs. 83.4, *p* = 0.025) than those referred by their Primary Care Provider (PCP). After the RFBHC establishment, there was no longer a statistically significant difference in overall wait time for SMP vs. PCP referred patients in either the Traditional Stream group (71.1 vs. 73.4, *p* = 0.738) or the RFBHC group (49.6 vs. 48.1, *p* = 0.720), as illustrated by Figure 5.

The patient experience survey had a response rate of 40.3% (148/367). Regarding question one, “Thinking of your most recent visit, how would you rate the length of time it took between your referral/screening mammogram to the time you were first seen at the clinic?”, 91.9% of respondents chose either “Good” (*n* = 24), “Very Good” (*n* = 30) or “Excellent” (*n* = 82). Regarding question two, “Thinking of your most recent visit, how would you rate your overall experience?”, 98.0% of respondents chose either “Good” (*n* = 8), “Very Good” (*n* = 24) or “Excellent” (*n* = 113).

## 4. Discussion

The RFBHC improved breast cancer diagnostic and treatment wait times when compared with pre-RFBHC and Traditional Stream groups. Additionally, the RFBHC improved regional compliance with national and provincial breast cancer standards, and resolved the diagnostic wait time disparity between screening mammography referrals and symptomatic patient referrals. Respondents to a RFBHC patient survey reported an overall positive clinic experience. This study illustrates that a Canadian BHC, which services rural-remote populations, can improve breast cancer services and wait times comparable to BHCs in larger Canadian communities [29,30].

The RFBHC achieved a mean 29.8-day reduction in overall breast cancer wait time when compared to pre-RFBHC patients. The expansion of RIH breast diagnostic imaging services, which occurred with the opening of the RFBHC, contributed to this improvement. However, post-RFBHC establishment, RFBHC patients experienced a mean 24 days reduced overall breast cancer wait time than Traditional Stream patients, indicating that the RFBHC had a positive impact on wait times independent of expanded imaging ser vices.

The reduced overall breast cancer wait time experienced by the RFBHC was primarily driven by a mean 21.3-day reduction in breast cancer diagnostic wait time when compared to the pre RFBHC group. Analysis of secondary endpoints revealed that this reduction occurred because of shorter wait times from presentation to imaging and from imaging to biopsy. The improved wait time from presentation to imaging was expected with the hiring of additional breast diagnostic imaging staff and the introduction of a nurse navigator. The improved wait time from imaging to biopsy was also expected with the introduction of same day US guided biopsy through the “triple assessment in one day” protocol.

Conversely, an 8.5-day shorter treatment interval for RFBHC patients when compared to pre-RFBHC patients was unexpected, as the RFBHC focus is on streamlining care to a breast cancer diagnosis rather than to treatment. Findings of a shorter treatment interval also contrast with other Canadian studies examining the impact of coordinated breast diagnostic services on surgical wait times. Both Webber et al. [24] and Racz et al. [30] reported relatively longer treatment wait times for patients receiving coordinated breast diagnostic services. A potential explanation for the shorter treatment time identified in the present study is the central surgical referral system used by the RFBHC, which was established independently but at the time as the RFBHC opening [58].

Traditional Stream patients experienced an 8.8-day diagnostic wait time reduction when compared with the pre-RFBHC group, likely secondary to the RIH medical imaging breast diagnostic staff expansion. Unfortunately, this reduction did not translate to an overall reduction in wait times for the Traditional Stream group when compared to the pre-RFBHC group. This finding presented a quality improvement opportunity. Changes have been made at RIH to ensure that Traditional Stream patients receive their diagnostic breast imaging in a similar time frame as RFBHC patients. Data collection to determine the impact of these changes is ongoing.

Establishment of the RFBHC improved regional adherence to Canadian Pan National and BC Provincial breast cancer wait time standards. However, this improvement was only statistically significant for the RFBHC group. The RFBHC improved adherence to the 2012 BC Provincial Health strategy recommendations. However, the region continues to not fully meet the recommendation that 90% of women receive their breast cancer diagnosis within 28 days of presentation, demonstrating the need for additional system changes to improve compliance with these standards.

The RFBHC was successful in rectifying the wait time disparity for Screening Mammography Program (SMP) and Primary Care Provider (PCP) referred patients. This was achieved by the expansion of RIH breast diagnostic services in addition to modifications in breast diagnostic imaging triaging guidelines.

Results of this study must be considered in light of study limitations. The study was limited to a single site, which limits its generalizability. The wait time data used for this study were initially collected as part of a QI project and do not include specific information on patient address or socioeconomic status. Therefore, it was not possible to evaluate the direct impact of the RFBHC on rural-remote and otherwise marginalized patients. Further studies on the impact of coordinated breast health services in small and less-densely populated Canadian regions would be beneficial. Data collection started at the time of the RFBHC establishment and wait times may change as the clinic develops. Finally, beyond a limited patient experience survey, this study did not fully address patient or provider experience which is an important piece of the clinic’s impact, especially for patients in rural-remote communities.

## 5. Conclusions

The establishment of a breast health clinic using the triple assessment model in a mid-sized Canadian center servicing urban and rural-remote communities resulted in (1) decreased wait times within the breast cancer diagnostic and treatment pathway, (2) improved compliance with national and provincial breast cancer wait time standards, and (3) reduction in the breast cancer diagnostic wait time disparity for SMP and PCP referred patients. This study contributes to the body of real-world evidence demonstrating that breast health clinics can substantially improve breast cancer diagnosis and treatment wait times.

## Figures and Tables

**Table 1:** Pan-Canadian and BC Provincial Wait Time Standards for Breast Cancer Diagnosis and Treatment.

Source	Milestone	Wait Time Standard	Target Compliance
Pan-Canadian Standards for Breast Cancer Surgery	Symptoms to Pathology	6 weeks	90%
	Pathology to Consult	2 weeks	90%
	Consult to Treatment	4 weeks	90%
Canadian National Breast Cancer Screening Strategy (Current BC Standard)	Symptoms to Pathology	7 weeks	90%
BC Provincial Breast Health Strategy Summary Report (Recommended BC Standard)	Symptoms to Pathology	4 weeks	90%

**Table 2:** Summary of study variables.

**Characteristics**	**Description**	**Pre-RFBHC**	**RFBHC** **Established:** **Traditional Stream**	**RFBHC** **Established:** **Referred to RFBHC**	**Full Cohort**
Age	Mean ± 95% CI	64.4 ± 1.3	64.2 ± 2.6	63.4 ± 2.1	64.2 ± 1.0
	Median (Q2 to Q3)	66.0 (56.0 to 72.0)	65.5 (54.5 to 74.0)	62.5 (57.0 to 70.0)	65.0 (57.0 to 72.0)
	Number of Patients	327	104	108	539
Location	Kamloops	222 (67.9%)	63 (60.6%)	77 (71.3%)	362 (67.2%)
	Outside of Kamloops	105 (32.1%)	41 (39.4%)	31 (28.7%)	177 (32.8%)
Source of Referral	Primary Care Provider	191 (59.0%)	89 (85.6%)	39 (36.1%)	321 (59.6%)
	Screening Mammography Program	134 (41.0%)	15 (14.4%)	69 (63.9%)	218 (40.4%)
Pathologic Diagnosis	Invasive Ductal Adenocarcinoma	251 (76.8%)	74 (71.2%)	74 (68.5%)	399 (74.0%)
	DCIS	48 (14.7%)	21 (20.2%)	14 (13.0%)	83 (15.4%)
	Lobular Carcinoma	28 (8.6%)	9 (8.7%)	20 (18.5%)	57 (10.6%)

**Table 3:** Comparative compliance with Pan-Canadian [53] and Provincial [54,55] breast cancer wait time standards between study groups.

**Standard**	**Milestone Interval**	**Pre-RFBHC**	**RFBHC Established:** **Traditional Stream**	**RFBHC Established:** **Referred to RFBHC**
Pan-Canadian Standards for Breast Cancer Surgery	Symptoms to Pathology (90% within 6 weeks)	203/327 (62.1%) Referent	74/104 (71.2%) *p* = 0.093	99/108 (91.7%) *p* ≤ 0.001
	Pathology to Consult (90% within 2 weeks)	231/327 (70.6%) Referent	61/104 (58.7%) *p* = 0.023	84/108 (77.8%) *p* = 0.151
	Consult to Treatment (90% within 4 weeks)	263/327 (80.4%) Referent	80/104 (76.9%) *p* = 0.441	96/108 (88.9%) *p* = 0.045
Canadian National Breast Cancer Screening Strategy (Current BC standard)	Symptoms to Pathology (90% within 7 weeks)	230/327 (70.3%) Referent	81/104 (77.9%) *p* = 0.135	102/108 (94.4%) *p* ≤ 0.001
BC Provincial Breast Health Strategy Summary Report (Recommended BC standard)	Symptoms to Pathology (90% within 4 weeks)	115/327 (35.2%) Referent	46/104 (44.2%) *p* = 0.097	89/108 (82.4%) *p* ≤ 0.001

## Data Availability

The data presented in this study are available on request from the corresponding author.

## References

[1] McKevitt E.C., Dingee C.K., Leung S.-P., Brown C.J., Van Laeken N.Y., Lee R., Kuusk U. (2017). Reduced Time to Breast Cancer Diagnosis with Coordination of Radiological and Clinical Care. Cureus.

[2] Pelletier L. (2012). Provincial Breast Health Strategy Summary Report.

[3] Flores-Balcázar C.H., Flores-Luna M.L., Villarreal-Garza C.M., Bargalló-Rocha J.E. (2020). Provider delay in treatment initiation and its influence on survival outcomes in women with operable breast cancer. Rep. Pract. Oncol. Radiother..

[4] Ho P.J., Cook A.R., Binte Mohamed Ri N.K., Liu J., Li J., Hartman M. (2020). Impact of delayed treatment in women diagnosed with breast cancer: A population-based study. Cancer Med..

[5] Yung R., Ray R.M., Roth J., Johnson L., Warnick G., Anderson G.L., Kroenke C.H., Chlebowski R.T., Simon M.S., Fung C. (2020). The association of delay in curative intent treatment with survival among breast cancer patients: Findings from the Women’s Health Initiative. Breast Cancer Res. Treat..

[6] Bleicher R.J. (2018). Timing and Delays in Breast Cancer Evaluation and Treatment. Ann. Surg. Oncol..

[7] Eriksson L., Bergh J., Humphreys K., Wärnberg F., Törnberg S., Czene K. (2018). Time from breast cancer diagnosis to therapeutic surgery and breast cancer prognosis: A population-based cohort study. Int. J. Cancer.

[8] Mansfield S.A., Abdel-Rasoul M., Terando A.M., Agnese D.M. (2017). Timing of Breast Cancer Surgery-How Much Does It Matter?. Breast J..

[9] Webber C., Jiang L., Grunfeld E., Groome P.A. (2017). Identifying predictors of delayed diagnoses in symptomatic breast cancer: A scoping review. Eur. J. Cancer Care.

[10] Richards M., Westcombe A., Love S., Littlejohns P., Ramirez A. (1999). Influence of delay on survival in patients with breast cancer: A systematic review. Lancet.

[11] McGarvey N., Gitlin M., Fadli E., Chung K.C. (2022). Increased healthcare costs by later stage cancer diagnosis. BMC Health Serv. Res..

[12] Blumen H., Fitch K., Polkus V. (2016). Comparison of Treatment Costs for Breast Cancer, by Tumor Stage and Type of Service. Am. Health Drug Benefits.

[13] Pineault P. (2007). Breast Cancer Screening: Women’s Experiences of Waiting for Further Testing. Oncol. Nurs. Forum.

[14] Boudreau R.M., McNally C., Rensing E.M., Campbell M.K. (2004). Improving the Timeliness of Written Patient Notification of Mammography Results by Mammography Centers. Breast J..

[15] Ganz P.A. (2000). Quality of Life Across the Continuum of Breast Cancer Care. Breast J..

[16] Thorne S.E., Harris S.R., Hislop T.G., Vestrup J.A. (1999). The Experience of Waiting for Diagnosis after an Abnormal Mammogram. Breast J..

[17] Biganzoli L., Cardoso F., Beishon M., Cameron D., Cataliotti L., Coles C.E., Bolton R.C.D., Trill M.D., Erdem S., Fjell M. (2020). The requirements of a specialist breast centre. Breast.

[18] Biganzoli L., Marotti L., Hart C.D., Cataliotti L., Cutuli B., Kühn T., Mansel R.E., Ponti A., Poortmans P., Regitnig P. (2017). Quality indicators in breast cancer care: An update from the EUSOMA working group. Eur. J. Cancer.

[19] Guler S.A., Güllüoğlu B.M. (2014). Quality Assurance in Breast Health Care and Requirement for Accreditation in Specialized Units. J. Breast Health.

[20] Wilson A., Marotti L., Bianchi S., Biganzoli L., Claassen S., Decker T., Frigerio A., Goldhirsch A., Gustafsson E., Mansel R. (2013). The requirements of a specialist Breast Centre. Eur. J. Cancer.

[21] Perry N., Broeders M., de Wolf C., Törnberg S., Holland R., von Karsa L. (2008). European guidelines for quality assurance in breast cancer screening and diagnosis. Fourth edition—Summary document. Ann. Oncol..

[22] Winchester D.P. (2016). The United States’ national accreditation program for breast centers: A model for excellence in breast disease evaluation and management. Chin. Clin. Oncol..

[23] Kaufman C.S., Shockney L., Rabinowitz B., Coleman C., Beard C., Landercasper J., Askew J.B., Wiggins D., Quality Initiative Committee (2010). National Quality Measures for Breast Centers (NQMBC): A Robust Quality Tool. Ann. Surg. Oncol..

[24] Webber C., Whitehead M., Eisen A., Holloway C., Groome P. (2020). Breast Cancer Diagnosis and Treatment Wait Times in Specialized Diagnostic Units Compared with Usual Care: A Population-Based Study. Curr. Oncol..

[25] Blackmore K.M., Weerasinghe A., Holloway C.M.B., Majpruz V., Mirea L., O’Malley F.P., Harris C.P., Hendry A., Hey A., Kornecki A. (2019). Comparison of wait times across the breast cancer treatment pathway among screened women undergoing organized breast assessment versus usual care. Can. J. Public Health.

[26] Jiang L., Gilbert J., Langley H., Moineddin R., Groome P.A. (2018). Breast cancer detection method, diagnostic interval and use of specialized diagnostic assessment units across Ontario, Canada. Health Promot. Chronic Dis. Prev. Can..

[27] Chiarelli A.M., Muradali D., Blackmore K.M., Smith C.R., Mirea L., Majpruz V., O’Malley F.P., Quan M.L., Holloway C.M. (2017). Evaluating wait times from screening to breast cancer diagnosis among women undergoing organised assessment vs usual care. Br. J. Cancer.

[28] Jiang L., Gilbert J., Langley H., Moineddin R., Groome P.A. (2015). Effect of specialized diagnostic assessment units on the time to diagnosis in screen-detected breast cancer patients. Br. J. Cancer.

[29] McKevitt E.C., Dingee C.K., Warburton R., Pao J.S., Brown C., Wilson C., Kuusk U. (2017). Coordination of Radiologic and Clinical Care Reduces the Wait Time to Breast Cancer Diagnosis. Curr. Oncol..

[30] Racz J., Holloway C., Huang W., Hong N.L. (2016). Improving Patient Flow and Timeliness in the Diagnosis and Management of Breast Abnormalities: The Impact of a Rapid Diagnostic Unit. Curr. Oncol..

[31] Baliski C., McGahan C.E., Liberto C.M., Broughton S., Ellard S., Taylor M., Bates J., Lai A. (2014). Influence of nurse navigation on wait times for breast cancer care in a Canadian regional cancer center. Am. J. Surg..

[32] Psooy B.J., Schreuer D., Borgaonkar J., Caines J.S. (2004). Patient navigation: Improving timeliness in the diagnosis of breast abnormalities. Can. Assoc. Radiol. J..

[33] Landercasper J., Linebarger J.H., Ellis R.L., Mathiason M.A., Johnson J.M., Marcou K.A., De Maiffe B.M., Jago G.S. (2010). A Quality Review of the Timeliness of Breast Cancer Diagnosis and Treatment in an Integrated Breast Center. J. Am. Coll. Surg..

[34] Castellanos M.R., Conte J., Fadel D.A., Raia C., Forte F., Ahern K., Smith M., ElSayeh D., Buchbinder S. (2008). Improving Access to Breast Health Services with an Interdisciplinary Model of Care. Breast J..

[35] Argenbright K., Anderson R.P., Senter M., Lee S.C. (2012). Breast Screening and Patient Navigation in Rural Texas Counties—Strategic Steps. Tex. Public Health J..

[36] Depke J.L., Boreen A., Onitilo A.A. (2015). Navigating the Needs of Rural Women with Breast Cancer: A Breast Care Program. Clin. Med. Res..

[37] Inrig S.J., Higashi R.T., Tiro J.A., Argenbright K.E., Lee S.J.C. (2017). Assessing local capacity to expand rural breast cancer screening and patient navigation: An iterative mixed-method tool. Eval. Program Plan..

[38] Inrig S.J., Tiro J.A., Melhado T.V., Argenbright K.E., Lee S.J.C. (2014). Evaluating a De-Centralized Regional Delivery System for Breast Cancer Screening and Patient Navigation for the Rural Underserved. Tex. Public Health J..

[39] Obeng-Gyasi S., Obeng-Gyasi B., Tarver W. (2022). Breast Cancer Disparities and the Impact of Geography. Surg. Oncol. Clin. N. Am..

[40] Sprague B.L., Ahern T.P., Herschorn S.D., Sowden M., Weaver D.L., Wood M.E. (2021). Identifying key barriers to effective breast cancer control in rural settings. Prev. Med..

[41] Williams F., Jeanetta S., James A. (2016). Geographical Location and Stage of Breast Cancer Diagnosis: A Systematic Review of the Literature. J. Health Care Poor Underserved.

[42] Youl P.H., Aitken J.F., Turrell G., Chambers S.K., Dunn J., Pyke C., Baade P.D. (2016). The Impact of Rurality and Disadvantage on the Diagnostic Interval for Breast Cancer in a Large Population-Based Study of 3202 Women in Queensland, Australia. Int. J. Environ. Res. Public Health.

[43] Yuan Y., Li M., Yang J., Elliot T., Dabbs K., Dickinson J.A., Fisher S., Winget M. (2016). Factors related to breast cancer detection mode and time to diagnosis in Alberta, Canada: A population-based retrospective cohort study. BMC Health Serv. Res..

[44] Williams F., Jeanetta S., O’Brien D., Fresen J. (2015). Rural-urban difference in female breast cancer diagnosis in Missouri. Rural Remote Health.

[45] Keeton K.M., Jones E.S., Sebastian S. (2014). Breast Cancer in Mississippi: Impact of Race and Residential Geographical Setting on Cancer at Initial Diagnosis. South. Med. J..

[46] Leung J., McKenzie S., Martin J., McLaughlin D. (2014). Effect of rurality on screening for breast cancer: A systematic review and meta-analysis comparing mammography. Rural Remote Health.

[47] John P.S., Menec V., Tate R., Newall N., O’Connell M., Cloutier D. (2021). Functional status in rural and urban adults: The Canadian Longitudinal Study on Aging. J. Rural Health.

[48] Kornelsen J., Khowaja A.R., Av-Gay G., Sullivan E., Parajulee A., Dunnebacke M., Egan D., Balas M., Williamson P. (2021). The rural tax: Comprehensive out-of-pocket costs associated with patient travel in British Columbia. BMC Health Serv. Res..

[49] Kornelsen J., Carthew C., Míguez K., Taylor M., Bodroghy C., Petrunia K., Roberts D. (2021). Rural citizen-patient priorities for healthcare in British Columbia, Canada: Findings from a mixed methods study. BMC Health Serv. Res..

[50] Wong S., Regan S. (2009). Patient perspectives on primary health care in rural communities: Effects of geography on access, continuity and efficiency. Rural Remote Health.

[51] Shuswap T.C., Health P.H., Status S., Care A. (2020). Health Service Delivery Area Profile.

[52] Nigam M., Nigam B. (2013). Triple Assessment of Breast—Gold Standard in Mass Screening for Breast Cancer Diagnosis. IOSR J. Dent. Med. Sci..

[53] Quan M., Finley C. (2019). Pan-Canadian Standards for Breast Cancer Surgery.

[54] Canadian Partnership against Cancer (2013). Report from the Evaluation Indicators Working Group: Guidelines for Monitoring Breast Cancer Screening Program Performance.

[55] Provincial Health Services Authority (2010). Breast Health Action Plan.

[56] Health Quality Ontario (2015). Primary Care Patient Experience Survey.

[57] Health Quality Ontario (2015). Primary Care Patient Experience Survey: Support Guide.

[58] Cha J., McKevitt E., Pao J.-S., Dingee C., Bazzarelli A., Warburton R. (2020). Access to surgery following centralization of breast cancer surgical consultations. Am. J. Surg..

